# Effects of osmolytes and macromolecular crowders on stable GAAA tetraloops and their preference for a CG closing base pair

**DOI:** 10.7717/peerj.4236

**Published:** 2018-02-13

**Authors:** Kaethe N. Leonard, Joshua M. Blose

**Affiliations:** Department of Chemistry and Biochemistry, State University of New York, The College at Brockport, Brockport, NY, United States of America

**Keywords:** Macromolecular crowding, GNRA, GAAA, Osmolytes, Nucleic acid structure, RNA folding, Polyethelyne glycol, Hairpins

## Abstract

Osmolytes and macromolecular crowders have the potential to influence the stability of secondary structure motifs and alter preferences for conserved nucleic acid sequences *in vivo*. To further understand the cellular function of RNA we observed the effects of a model osmolyte, polyethylene glycol (PEG) 200, and a model macromolecular crowding agent, PEG 8000, on the GAAA tetraloop motif. GAAA tetraloops are conserved, stable tetraloops, and are critical participants in RNA tertiary structure. They also have a thermodynamic preference for a CG closing base pair. The thermal denaturation of model hairpins containing GAAA loops was monitored using UV-Vis spectroscopy in the presence and absence of PEG 200 or PEG 8000. Both of the cosolutes tested influenced the thermodynamic preference for a CG base pair by destabilizing the loop with a CG closing base pair relative to the loop with a GC closing base pair. This result also extended to a related DNA triloop, which provides further evidence that the interactions between the loop and closing base pair are identical for the d(GCA) triloop and the GAAA tetraloop. Our results suggest that in the presence of model PEG molecules, loops with a GC closing base pair may retain some preferential interactions with the cosolutes that are lost in the presence of the CG closing base pair. These results reveal that relatively small structural changes could influence how neutral cosolutes tune the stability and function of secondary structure motifs *in vivo*.

## Introduction

The cellular environment is a chemically complex medium compared to the dilute solution conditions used in many typical biochemical analyses. Large macromolecules crowd the cell and can concentrate to nearly 40% (w/v) (Zimmerman & Trach, 1991; Ellis, 2001). In addition, osmolytes, which are small, chemically diverse neutral cosolutes such as amino acids and polyols, accumulate in response to environmental stresses such as changes in temperature, pressure, and salinity (Bolen, 2001; Burg & Ferraris, 2008). Osmolyte concentration is dependent on the type of cell and the type of stress experienced (Yancey et al., 1982; Yancey, 2005; Burg & Ferraris, 2008; Khan et al., 2010). Despite their chemical diversity, protecting osmolytes stabilize proteins via the common mechanism of preferential exclusion (Bolen, 2001; Burg & Ferraris, 2008). Protecting osmolytes are excluded from protein surfaces which destabilizes the unfolded state relative to the folded state. Thus, osmolyte concentrations are carefully regulated to preserve protein structure (Record et al., 1998).

In contrast to their effects on protein structures, the effects of macromolecular crowders and osmolytes on nucleic acid structures have been shown to be rather complex and have significant dependencies on the chemical and structural properties of both the nucleic acid and the cosolute (Spink & Chaires, 1999; Lambert & Draper, 2007; Downey et al., 2007; Nakano et al., 2012; Nakano et al., 2008; Nakano et al., 2009; Kilburn et al., 2010; Lambert, Leipply & Draper, 2010; Hart, Harris & Testa, 2010; Knowles et al., 2011; Blose et al., 2011; Pramanik et al., 2011; Gu et al., 2013). Osmolytes and crowders have been shown repeatedly to destabilize some secondary structures (Spink & Chaires, 1999; Lambert & Draper, 2007; Nakano et al., 2008; Nakano et al., 2012; Lambert, Leipply & Draper, 2010; Hart, Harris & Testa, 2010; Knowles et al., 2011; Blose et al., 2011; Pramanik et al., 2011; Gu et al., 2013; Strulson et al., 2013) while either stabilizing or destabilizing multi-stranded or more complex, higher-order structures (Spink & Chaires, 1999; Lambert & Draper, 2007; Downey et al., 2007; Lambert, Leipply & Draper, 2010; Strulson et al., 2014). Some osmolytes and macromolecular crowders have also been shown to facilitate folding and function of catalytic RNA (Nakano et al., 2009; Nakano et al., 2014; Kilburn et al., 2010; Strulson et al., 2013). The effects observed for nucleic acid folding and function in the presence of these neutral cosolutes arise from a complex combination of changes in hydration, in preferential interactions between nucleic acids and osmolytes, in excluded volume, and in solution dielectric constant. In order to add to our understanding of how neutral cosolutes influence the folding and function of RNA, we chose to study their influence on the stability of model hairpins containing GAAA tetraloops.

The most common RNA hairpin loops are tetraloops, and approximately 50% of the hairpin structures in ribosomal RNA contain tetraloops (Woese, Winker & Gutell, 1990; Wolters, 1992; Gutell, 1993). In addition, loops of the GNRA family, where N is any nucleotide and R is a purine, along with UNCG motif are some of the most common and most stable (Woese, Winker & Gutell, 1990; Wolters, 1992; Gutell, 1993). GNRA loops possess increased thermodynamic stability in the presence of a CG closing base pair (Antao & Tinoco Jr, 1992; Moody, Feerrar & Bevilacqua, 2004; Blose et al., 2009; Blose, Lloyd & Bevilacqua, 2009) and have been extensively studied (Varani, 1995; Bevilacqua & Blose, 2008; Bottaro & Lindorff-Larsen, 2017) due to their participation in critical RNA tertiary interactions, which have been reviewed elsewhere (Butcher & Pyle, 2011; Fiore & Nesbitt, 2013; Bisaria & Herschlag, 2015). Moreover, GNRA loops are now used to facilitate solving of RNA crystal structures and are benchmark structures for computational predictions of RNA structure, folding, and dynamics (Coonrod, Lohman & Berglund, 2012; Kührová et al., 2013; Chen & Garcia, 2013; Haldar et al., 2015).

Given the significant role for GNRA loops in RNA folding, they make an excellent system to further the investigations of the influence of cosolutes on RNA secondary structures. Here we have used model RNA hairpins containing the common GAAA version of a GNRA tetraloop to determine not just the influence of cosolutes on the stability of the loop, but also on the thermodynamic preference for a CG closing base pair. This work would serve to complement prior studies on GNRA loops that focused on how cosolutes influence the tertiary interactions of the loops rather than the secondary structures (Dupuis, Holmstrom & Nesbitt, 2014; Holmstrom, Dupuis & Nesbitt, 2015). By monitoring the denaturation of the model hairpins in the presence and absence model PEG cosolutes, we elucidated the effects of the cosolutes on the loop itself and on the preference for a CG closing base pair. We found that polyethylene glycols significantly destabilized the tetraloop with a CG closing base pair relative to a tetraloop with a GC closing base pair. This effect was linear with increasing PEG 200 concentration and suggested that cosolutes may retain preferential interactions with the loop nucleobases with a GC closing base pair.

## Experimental

### Hairpin synthesis

The RNA hairpins and associated DNA hairpins used in this study were synthesized by Integrated DNA Technologies (Coralville, IA, USA) and were diluted to a concentration of 1 mM using 10 mM Tris, 0.1 mM EDTA pH 7.5 buffer for storage. The RNA hairpin sequences studied were 1cg: 5′-GG*A***C**GAAA**G***U*CC-3′; 1gc: 5′-GG*A***G**GAAA**C***U*CC-3′; 2cg: 5′-G*AA***C**GAAA**G***UU*C-3′; and 2gc: 5′-G*AA***G**GAAA**C***UU*C-3′. Each hairpin contains a GAAA tetraloop (underlined), a CG or GC closing base pair (bolded), and an additional three base pairs in the stem that contains one or two AU pairs (italicized). The DNA hairpin sequences studied were d2cg: 5′-G*AA***C**GCA**G***TT*C-3′; and d2gc: 5′-G*AA***G**GCA**C***TT*C-3′. In this case both sequences contain a DNA triloop (underlined). Thus, sequences are labeled according to whether the loop closing base pair is a CG or GC pair and if the stem contains one or two AU or AT pairs. Two different stems were utilized to account for non-nearest-neighbor effects that potentially could have contributed to the observed trends in stability for the GAAA tetraloops. The stem with two AT pairs also helped lower the *T*_*M*_ of the DNA hairpin to improve upper baselines in the denaturation studies as well as to compare with previous experiments (Blose, Lloyd & Bevilacqua, 2009). This is also why the DNA loop sequence of GCA was used instead of GAA (Blose, Lloyd & Bevilacqua, 2009).

### Preparation of cosolute solutions

Forty percent (w/v) solutions of cosolutes in 10 mM sodium phosphate pH 7 were prepared. Both the masses and volumes of solution components measured during solution preparation so that the concentration of the solutions could be expressed in additional concentration units, such as molal (*m*) as needed for comparison between studies. PEG 200 was used as a model osmolyte as it has been shown to influence the preference for a CG closing pair for related stable RNA hairpins (Whittum & Blose, 2017), and PEG 8000 was used as a larger cosolute and potential crowding agent.

### Thermal denaturation of hairpins

The nucleic acid concentrations of the denatured samples ranged from 3 to 45 µM. All melts contained 10 mM sodium phosphate pH 7 and 0–40% (w/v) osmolyte. Thermal denaturation of the model hairpins was performed using an Evolution 260 Bio spectrophotometer or a Chirascan CD spectrophotometer in absorbance mode. Sample absorbance was recorded at 260 and 280 nm for the RNA hairpins and 260 nm for the DNA hairpins to compare to the literature (Blose et al., 2009; Blose, Lloyd & Bevilacqua, 2009). The absorbance was recorded over a temperature range of 10 °C–90 °C using a temperature ramp of 1 °C min^−1^ as needed to obtain baselines for fits. Data were fit to a two-state model using sloping baselines and analyzed using a Levenberg-Marquadt algorithm for nonlinear curve fitting in QtiPlot v. 0.9.8.3 (http://www.qtiplot.com/).

### Thermodynamic analysis

**Figure 1:**
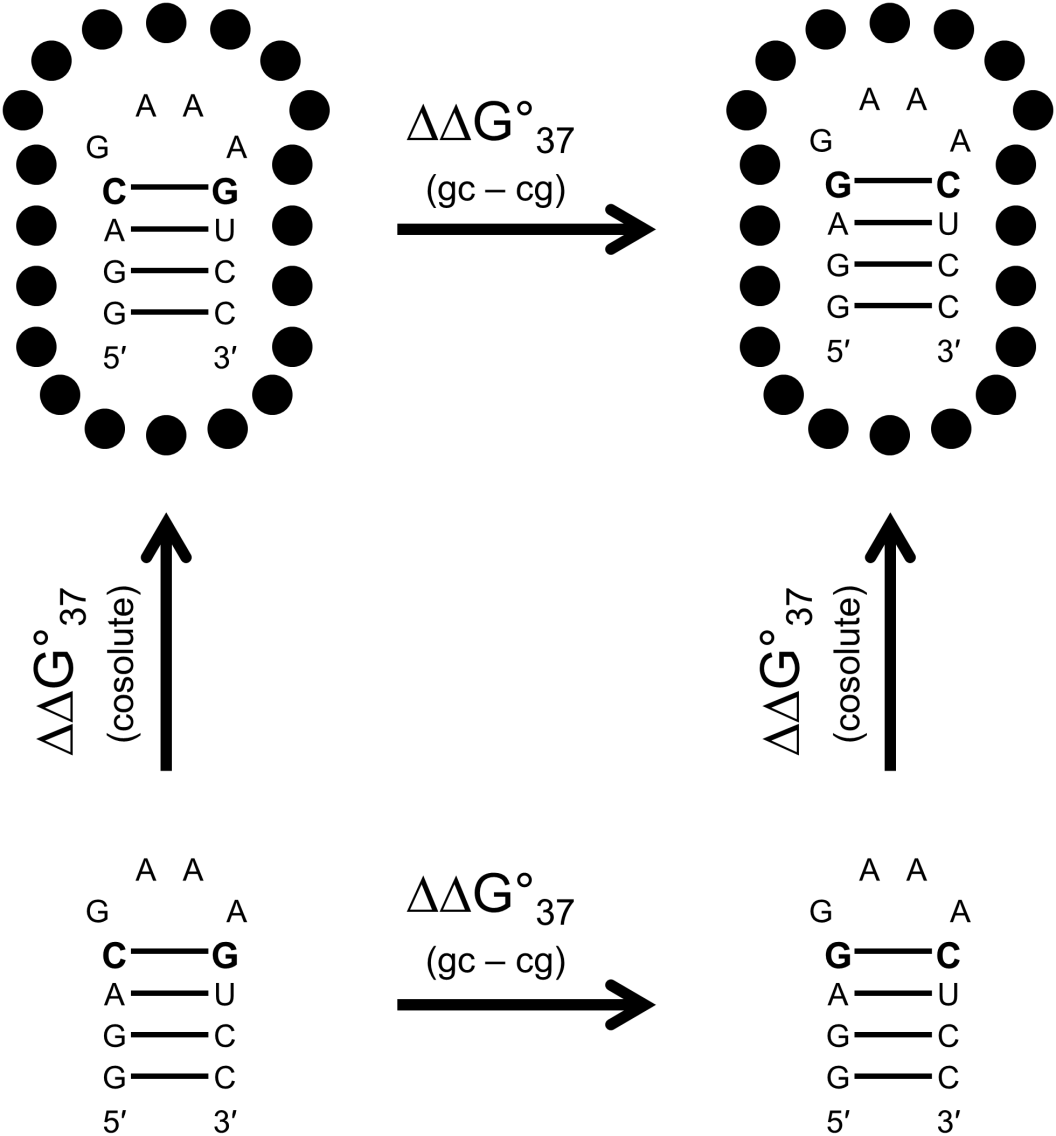
Thermodynamic analysis of GAAA hairpins. The hairpin structures are for sequence 1cg and 1gc, respectively. The black circles are a general representation of cosolutes in solution that could interact with the hairpin. Free energy values are described in detail via Eqs. (1)–(4) in the Materials and Methods. ΔΔG°_37(cosolute)_ is the change in the folding free energy for a sequence in a cosolute background. ΔΔG°_37(cosolute)_ quantifies the change in the folding free energy when a GC closing base pair replaces a CG closing base pair. This value can be adjusted to be specific to the GAAA tetraloop by accounting for the difference in stem stacking parameters ΔΔG°_37(loop)_. ΔΔG°_37(loop)_ values can be compared from in the presence and absence of cosolutes to determine the cosolute influence on loop stability ΔΔΔG°_37(loop,cosolute)_.

Thermodynamic parameters and uncertainties for hairpin folding are from the average and standard deviations of data from at least three independently prepared samples. The *T*_*M*_ was found to be independent of nucleic acid strand concentrations as expected. Previous studies on these sequences have shown that under the relatively low nucleic acid and buffer concentrations used in this study that our model sequences disfavor duplex formation and adopt the hairpin structure via intramolecular folding (Blose et al., 2009; Blose, Lloyd & Bevilacqua, 2009). To determine the influence of cosolutes on the stability of the stable hairpin loops and the thermodynamic preference for a CG closing base pair, ΔG°_37_ values were compared among sequences and solution conditions as modeled in Fig. 1. The change in folding stability due to the presence of a cosolute was quantified by

(1) \begin{eqnarray*}\Delta \Delta \mathrm{G}{\textdegree }_{\mathrm{37(cosolute)}}=\Delta \mathrm{G}{\textdegree }_{\mathrm{37(cosolute)}}-\Delta \mathrm{G}{\textdegree }_{\mathrm{37(nocosolute)}}\end{eqnarray*}

where ΔG°_37(cosolute)_ − ΔG°_37(nocosolute)_ are for the folding of the same hairpin sequence in the presence and absence of PEG 200 or PEG 8000, respectively. The change in folding stability due to the change of CG to a GC closing base pair was determined by

(2) \begin{eqnarray*}\Delta \Delta \mathrm{G}{\textdegree }_{\mathrm{37(gc- cg)}}=\Delta \mathrm{G}{\textdegree }_{\mathrm{37(gc)}}-\Delta \mathrm{G}{\textdegree }_{\mathrm{37(cg)}}\end{eqnarray*}

where ΔG°_37(gc)_ and ΔG°_37(cg)_ are for the folding of a hairpin with a GC and a CG closing base pair, respectively, under the same solution conditions. Equation (3) was used to determine the influence of the closing base pair change directly on the nucleic acid loops,

(3) \begin{eqnarray*}\Delta \Delta \mathrm{G}{\textdegree }_{37\mathrm{(loop)}}=\Delta \Delta \mathrm{G}{\textdegree }_{\mathrm{37(gc- cg)}}-0.16 {\text{kcal mol}}^{-1}\end{eqnarray*}

where the small factor of 0.16 kcal mol^−1^ accounts for the contribution of the change in stacking of the GC compared to the CG (Blose et al., 2009). Finally, the change in loop stability due to the switch from a CG to a GC closing base pair in the presence of a cosolute as compared to dilute buffer conditions is quantified in Eq. (4).

(4) \begin{eqnarray*}\Delta \Delta \Delta \mathrm{G}{\textdegree }_{\mathrm{37(loop,cosolute)}}=\Delta \Delta \mathrm{G}{\textdegree }_{37\mathrm{(loop,cosolute)}}-\Delta \Delta \mathrm{G}{\textdegree }_{37\mathrm{(loop,nocosolute)}}.\end{eqnarray*}

The ΔΔΔG°_37(loop,cosolute)_ parameter quantifies how PEG 200 or PEG 2000 effects the stability of the loop motif based on the selection of the loop closing base pair, and reveals both how the sequence preference may be influenced by cosolute and how small changes in sequence may influence cosolute-tuning of loop stability.

## Results and Discussion

### The CG closing base pair is thermodynamically preferred with both stems

Thermal denaturation experiments were performed for RNA hairpins containing a CG or GC closing base pair in the context of stem 1 or stem 2, which contain one or two AU pairs respectively. Parameters determined directly from the fit of these data along with uncertainties are found in Table 1, whereas further analysis along with the sum of squares propagated error (Harris, 2003) are summarized in Table 2. The comparison of hairpin folding with two different stems was performed to determine if non-nearest-neighbor effects influenced relative loop stability of GAAA loops with a CG versus a GC closing base pair in our model hairpins. It has been shown for related, stable GNRA loops that in addition to the closing base pair, the first non-nearest neighbor and to a lesser extent the second non-nearest neighbor to the tetraloop can affect loop stability (Sheehy, Davis & Znosko, 2010). For stem 1 the second non-nearest neighbor is a GC, and for stem 2 the second non-nearest neighbor is an AU. However, we only see minor effects on loop thermodynamics. Under our buffer conditions ΔΔG°_37(loop)_ is 1.47 kcal mol^−1^ and 1.36 kcal mol^−1^ for stem 1 and stem 2, respectively (Table 2, Column 4). Second non-nearest-neighbors have also been shown to have no significant on the stability of a stable DNA triloop d(GCA) in 10 mM sodium phosphate buffer (Blose, Lloyd & Bevilacqua, 2009). Moreover, our value of ΔΔG°_37(loop)_ agrees with previous work (1.33 vs. 1.38 kcal mol^−1^) on this model system (Blose, Lloyd & Bevilacqua, 2009). Thus, our observations suggest that the changes we observe in loop stability are not influenced by the stem outside of the closing base pair.

**Table 1:** Thermodynamic parameters for hairpin folding from denaturation curves.

Cosolute^a^/Hairpin	*T*_*M*_ (°C)	ΔH° (kcal mol^−1^)	ΔS° (e.u.)	ΔG°_**37**_^b^ (kcal mol^−1^)
**RNA hairpins**				
*No cosolute*				
1cg	67.9 ± 0.6	−42.2 ± 1.0	−123.7 ± 3.1	−3.82 ± 0.04
1gc	56.4 ± 0.4	−37.1 ± 0.9	−112.6 ± 2.2	−2.19 ± 0.05
2cg	53.7 ± 0.4	−37.0 ± 1.0	−113.3 ± 3.0	−1.89 ± 0.05
2gc	41.2 ± 0.5	−28.2 ± 1.6	−89.7 ± 5.0	−0.37 ± 0.04
*PEG 200*				
1cg	54.8 ± 0.6	−36.8 ± 1.3	−112.3 ± 4.1	−2.00 ± 0.05
1gc	46.4 ± 0.5	−34.3 ± 1.8	−107.7 ± 5.9	−1.00 ± 0.05
2cg	39.8 ± 0.5	−29.5 ± 1.4	−94.1 ± 4.6	−0.26 ± 0.04
2gc	30.2 ± 0.6	−30.7 ± 1.1	−101.3 ± 3.6	0.69 ± 0.06
*PEG 8000*				
1cg	64.7 ± 0.3	−35.8 ± 0.8	−106.9 ± 2.5	−2.93 ± 0.06
1gc	55.0 ± 0.4	−34.3 ± 1.3	−104.5 ± 4.0	−1.87 ± 0.04
**DNA hairpins**				
*No cosolute*				
d2cg	68.9 ± 0.5	−29.9 ± 0.9	−87.3 ± 2.6	−2.80 ± 0.05
d2gc	48.9 ± 0.4	−23.4 ± 0.7	−72.5 ± 2.3	−0.87 ± 0.03
*PEG 200*				
d2cg	45.7 ± 0.5	−27.3 ± 0.9	−85.6 ± 2.8	−0.74 ± 0.04
d2gc	30.2 ± 0.3	−26.3 ± 0.8	−86.6 ± 2.7	0.59 ± 0.03

**Notes.**

a Cosolute concentrations are 40% (w/v).

b Values are reported to two decimal places to prevent rounding errors.

**Table 2:** Thermodynamic analysis of the effect of the closing base pair and cosolutes on GAAA and d(GCA) hairpin stability.

Cosolute^a^/ Hairpin	ΔG°_37_^b^ (kcal mol^−1^)	ΔΔG°_37(gc−cg)_^c^ (kcal mol^−1^)	ΔΔG°_37(loop)_^c^ (kcal mol^−1^)	ΔΔG°37_(cosolute)_^c^ (kcal mol^−1^)	ΔΔΔG°_37(loop,cosolute)_^c^ (kcal mol^−1^)
**RNA hairpins**					
*No Cosolute*					
1cg	−3.82 ± 0.04				
1gc	−2.19 ± 0.05	1.63 ± 0.06	1.47 ± 0.06		
2cg	−1.89 ± 0.05				
2gc	−0.37 ± 0.04	1.52 ± 0.06	1.36 ± 0.06		
*PEG 200*					
1cg	−2.00 ± 0.05			1.82 ± 0.06	
1gc	−1.00 ± 0.05	1.00 ± 0.07	0.84 ± 0.07	1.19 ± 0.07	0.63 ± 0.09
2cg	−0.26 ± 0.04			1.63 ± 0.06	
2gc	0.69 ± 0.06	0.95 ± 0.07	0.79 ± 0.07	1.06 ± 0.07	0.57 ± 0.09
*PEG 8000*					
1cg	−2.93 ± 0.06			0.89 ± 0.07	
1gc	−1.87 ± 0.04	1.06 ± 0.07	0.90 ± 0.07	0.32 ± 0.06	0.57 ± 0.09
**DNA hairpins**					
*No cosolute*					
d2cg	−2.80 ± 0.05				
d2gc	−0.87 ± 0.03	1.93 ± 0.06	1.77 ± 0.06		
*PEG 200*					
d2cg	−0.74 ± 0.04			2.06 ± 0.07	
d2gc	0.59 ± 0.03	1.33 ± 0.05	1.17 ± 0.05	1.46 ± 0.04	0.60 ± 0.08

**Notes.**

a Osmolyte concentrations are 40% (w/v).

b Uncertainties are the standard deviations of data.

c Error was propagated using the sum-of-squares rule for subtraction.

### Model cosolutes destabilize hairpins with stable GAAA tetraloops

In order to add to the understanding of how osmolytes and crowders might influence the stability of the GAAA tetraloops we utilized presence and absence of polyethylene glycol (PEG) 200 and PEG 8000. We chose polyethylene glycols as they have been used extensively as neutral cosolutes to study a wide variety of nucleic acid structures including hairpins, duplexes, triplexes, and quadruplexes, and Z-DNA (Spink & Chaires, 1999; Spink, Garbett & Chaires, 2007; Miller et al., 2010; Knowles et al., 2011; Pramanik et al., 2011; Nakano et al., 2012; Gu et al., 2013; Strulson et al., 2013; Leamy, Yennawar & Bevilacqua, 2017). Moreover, it was found that PEG 200 influences not just the stability of but the preference for the CG closing base pair in UUCG tetraloops (Whittum & Blose, 2017).

**Figure 2:**
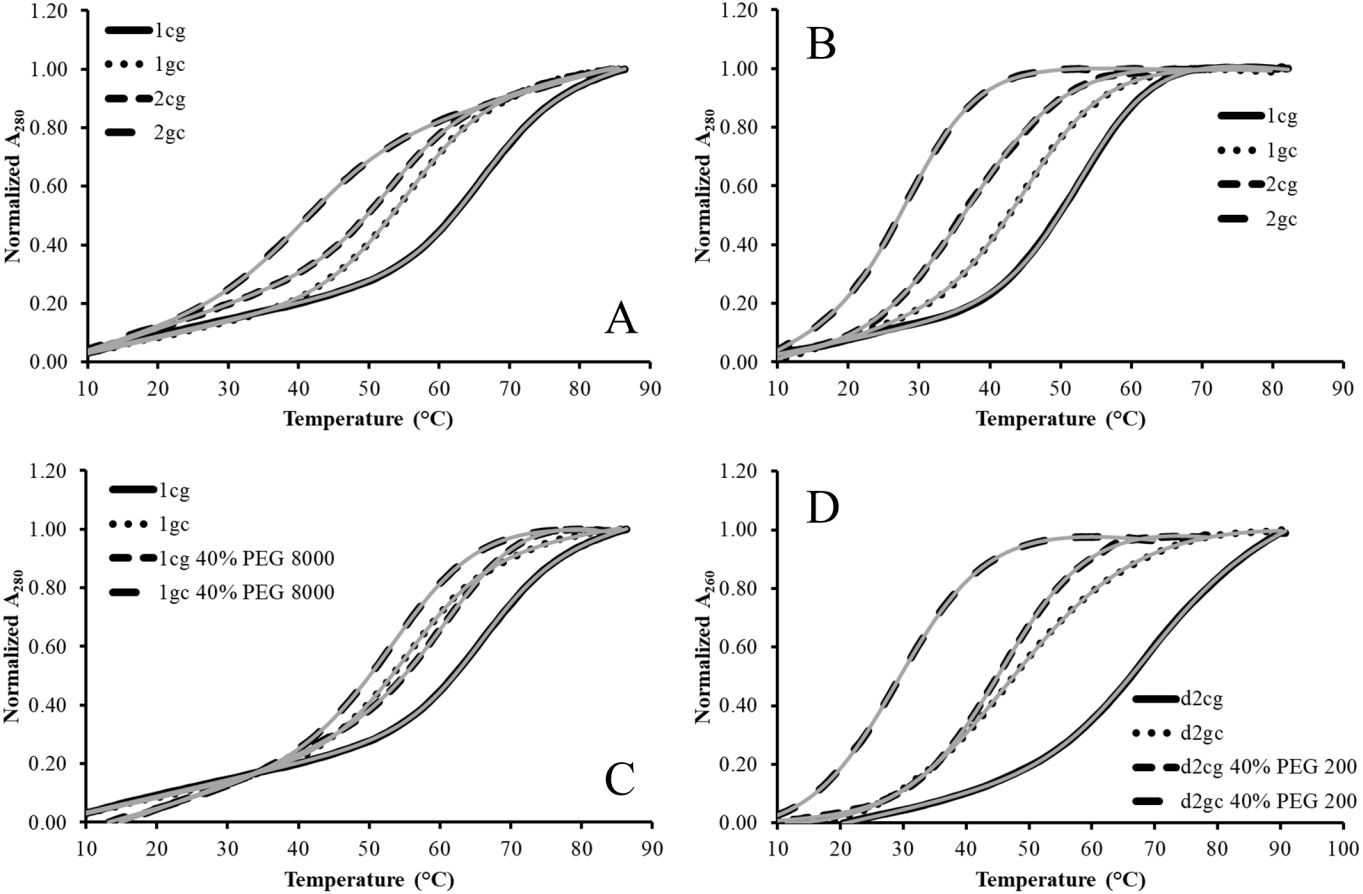
Representative absorbance vs temperature curves from thermal denaturations of model RNA and DNA hairpins. (A) Shows normalized A_280_ versus temperature for 1cg, 1gc, 2cg, and 2gc in the absence of cosolutes. (B) Shows the normalized A_280_ for 1cg − 2cg in 40% PEG 200. (C) Shows the normalized A_280_ for 1cg and 1gc in the presence and absence of 40% PEG 8000. (D) Shows the normalized A_260_ for d2cg and d2gc in the presence and absence of 40% PEG 200. Data was separated into (A–D) for clarity of the curves. For each curve, the fit to the two-state folding model is provided in gray. The qualitative shift to lower temperatures with a GC closing base pair shows the preference for the CG closing base pair, and the same shift to lower temperatures in the presence of cosolutes qualitatively shows that they destabilize all the hairpins tested.

Denaturation experiments were carried out for the RNA hairpins in the presence and absence of 40% (w/v) neutral cosolute and example denaturation curves are shown in Fig. 2. The cosolute concentration was chosen as it has been used in previous studies of cosolutes and nucleic acids as a high concentration limit for osmolytes and macromolecular crowders, and is representative of the high cellular concentration range for osmolytes (Khan et al., 2010). For the RNA hairpins, the presence of PEG 200 destabilizes the structures as can be seen qualitatively with the shift of the denaturation curves to lower temperatures (Fig. 2) and quantitatively in the positive values for ΔΔG°_37(cosolute)_ (Table 2). PEG 200 destabilized the 2cg and 2gc hairpins to nearly the same extent as for the 1cg and 1gc hairpins with differences of 0.19 and 0.13 kcal mol^−1^ for the cg and gc sequences, respectively. This provides further evidence that that second non-nearest neighbor effects are minor for the hairpins.

The destabilizing effects of PEG 200 on the GAAA loops are large and significant as ΔΔG°_37(cosolute)_ values exceeded 1 kcal mol^−1^ for all GAAA hairpin sequences. This is especially notable when the PEG 200 destabilization of the GAAA hairpin is compared to the nonprotecting osmolyte denaturation of a hairpin with an unstructured loop. For example, the PEG 200 destabilization of the 1cg hairpin is 1.5 times that for urea destabilization of a hairpin with an unstructured tetraloop and a cg closing base pair when compared on a per molal basis (0.59 kcal mol m^−1^ vs. 0.38 kcal mol m^−1^) (Lambert & Draper, 2007). Although small differences in concentrations of monovalent cations present in the experimental solutions may contribute to this observation, the major contributing factor to the destabilization of the GAAA hairpins is likely the significant stacking and hydrogen bonding present in the GAAA loop that is absent in the less stable tetraloop (Deng & Cieplak, 2010). These additional interactions present in the folded tetraloop would consequently limit the interactions of PEG 200 with the nucleobases as compared to the unfolded state.

The addition of PEG 8000 also destabilized the 1cg and 1gc hairpins, ΔΔG°_37(cosolute)_ (Table 2), but 0.9–1 kcal mol^−1^ less than for PEG 200. It has been found for a DNA hairpin that as the size of a PEG-based cosolute increases, direct interactions between the DNA hairpin and cosolute decrease and excluded volume effects increase (Knowles et al., 2011). Thus, the destabilization observed due to larger molecular weight PEG molecules is less than that for smaller molecular weight PEG molecules (Knowles et al., 2011). Given the destabilization for PEG 8000 is less than for PEG 200, especially for 1gc, we did not further purse its effects on the stem 2 hairpins.

### Model cosolutes preferentially destabilizes GAAA loops with a CG closing base pair

Both PEG 200 and PEG 8000 preferentially destabilize the RNA tetraloops with a CG closing base pair relative to those with a GC closing base pair as quantified by ΔΔΔG°_37(loop,cosolute)_ (Table 2, column 6). The preferential destabilization with a CG closing base pair decreases the free energy difference between the CG and GC loops by over 40% when one compares ΔΔΔG°_37(loop,cosolute)_ to ΔΔG°_37(loop)_. The value of ΔΔΔG°_37(loop,cosolute)_ is the same for both stem 1 and stem 2 within error for PEG 200, and more remarkably, it is the same within error in the presence of PEG 8000. Even though the overall destabilization of the RNA hairpins by PEG 8000 is significantly less than that for PEG 200, the difference in destabilization between sequences with CG and GC closing base pairs is the same within error. Moreover, the magnitude of ΔΔΔG°_37(loop,cosolute)_ when expressed on a per molal basis (∼0.20 kcal mol^−1^ m ^−1^) is on the same order of magnitude as the actual *m*-value for other cosolutes such as glycerol for an unstructured RNA loop (Lambert & Draper, 2007). This result suggests that the influence of the model cosolutes on the closing base pair preference is significant.

It has been suggested previously that although the loops are structurally dissimilar that the UUCG and GAAA loops mimic each other in how they present functional groups to and interact with their closing base pairs (Blose et al., 2009). PEG 200 destabilizes UUCG loops to a greater extent than GAAA loops, and the destabilization by PEG 200 is approximately 1.0 kcal mol^−1^ more for a UUCG loops than for GAAA loops with the same stem. For example, 1cg with a UUCG loop was destabilized by 2.84 kcal mol^−1^ by 40% PEG 200 (Whittum & Blose, 2017), but 1cg with a GAAA loop was destabilized by 1.82 kcal mol^−1^ (Table 2). Although the destabilization of the GAAA loops is less than observed for the UUCG loops, the relative impact on the preference for a CG closing base pair is similar. The ΔΔΔG°_37(loop,cosolute)_ values for GAAA loops are about 75% of that for UUCG. For example, ΔΔΔG°_37(loop,cosolute)_ is 0.63 kcal mol^−1^ for GAAA with the 1 cg stem whereas it is 0.80 kcal mol^−1^ for UUCG with the 1 cg stem (Whittum & Blose, 2017). This difference is just outside of the error on the two measurements, but this similarity in response of the closing base pair preference suggests that while UUCG and GAAA are structurally dissimilar, they are similar in how they interact with their closing base pairs, and in how osmolytes influence that preference.

**Figure 3:**
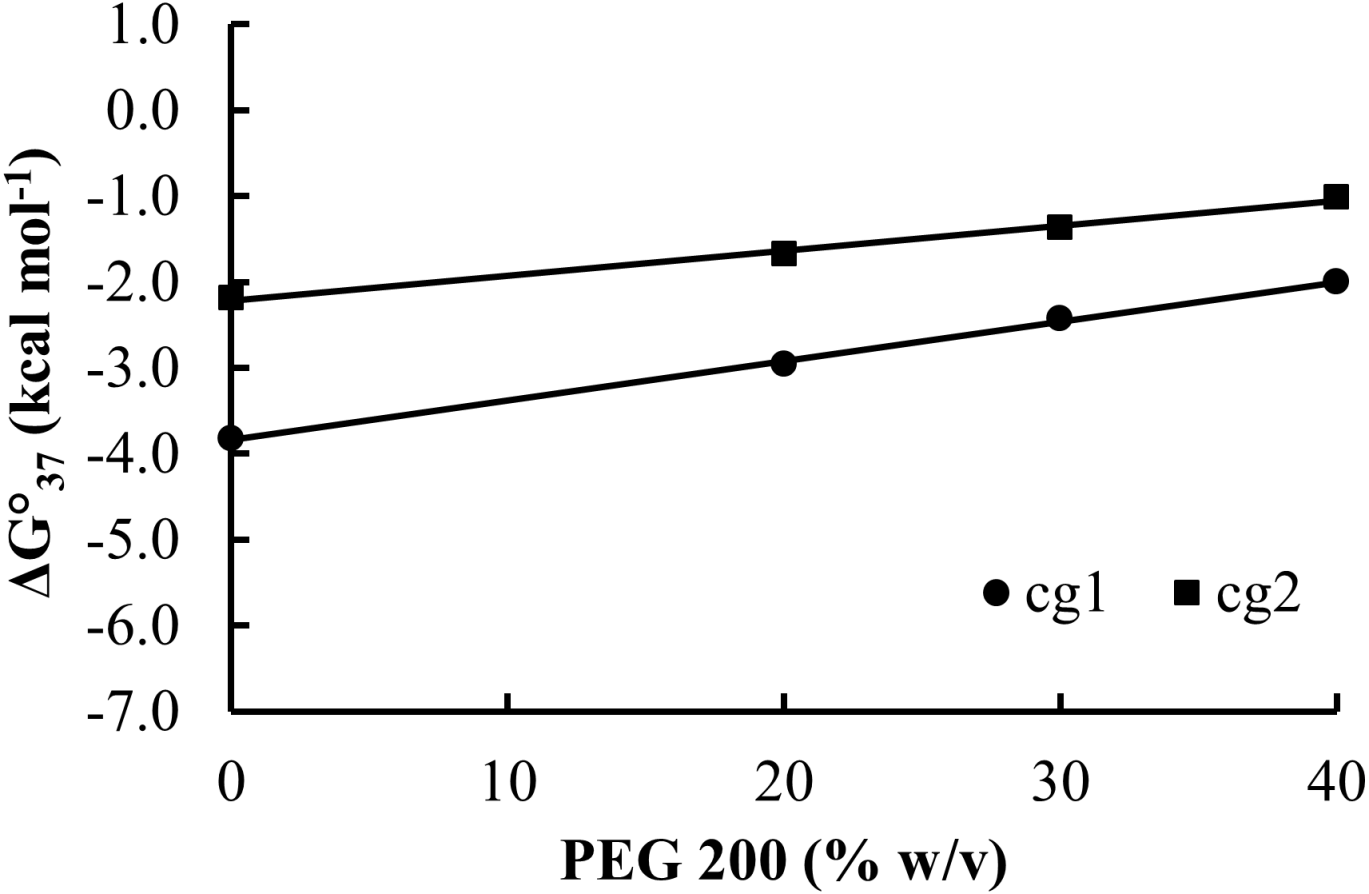
Linear relationships between thermodynamic parameters for hairpin folding and PEG 200 concentration. The free energy of folding for 1cg and 1gc, ΔG°_37(melt)_, is plotted as a function of PEG 200 concentration (w/v). The equations for the linear fits are *y* = 0.046*x* − 3.83 for 1cg and *y* = 0.029*x* − 2.21 for 1gc. The *R*^2^ values for the linear fits are 0.9984 and 0.994 for 1cg and 1gc, respectively.

To further investigate the difference in destabilization of the GAAA loops, additional PEG 200 concentrations were utilized for the representative 1cg and 1gc sequences and a plot of ΔG°_37_ vs. PEG 200% with data for 1cg and 1gc is provided in Fig. 3. There are distinctly linear trends in ΔG°_37_ with increasing PEG 200 concentration for both 1cg and 1gc (*R*^2^ > 0.9940) suggesting that the stability of the loops via PEG 200 is linearly tunable even at high at the highest cosolute concentrations.

### PEG 200 preferentially destabilizes d(GNA) Triloops with a CG closing base pair

Previous work on stable DNA triloops from the d(GNA) family has suggested that due to the similar interactions of the loop with its closing that this motif is portable across polymer type and loop size (Blose, Lloyd & Bevilacqua, 2009). Previously, analysis of the loop-closing base pair interaction included nucleotide and functional group modifications as well examining the salt dependence of the folding energies. Now, our PEG 200 comparisons add to this observation. If we compare ΔΔΔG°_37(loop,cosolute)_ for d(GNA) to that from the GNRA the values agree well within error (Table 2). This result suggests that in spite of differences in polymer type and properties that the interactions between the loops and closing base pairs is similar and that neutral cosolutes will tune stability based on those structural similarities.

### PEG 200 preferential destabilization is likely due to differences in the folded hairpin structure

It has been shown previously that cosolutes generally destabilize nucleic acid secondary structure via preferential interactions of the cosolute with the nucleobases in the unfolded state (Lambert & Draper, 2007; Knowles et al., 2011), and differences in observed m-values are then due to differences in the chemistry of specific interactions of the cosolutes with given sequences. For example, betaine prefers G-C pairs, urea prefers A-T pairs, whereas polyethylene glycols have previously shown no distinct sequence preference (Nordstrom et al., 2006; Spink, Garbett & Chaires, 2007; Knowles et al., 2011; Schwinefus et al., 2013). In determining these dependences, large global changes are often made; whereas for our RNA hairpin model system our sequence changes are small. The change from one to two AU pairs in the stem has no large, observable influence on the preference for a CG closing base pair by either of the RNA or DNA model systems. The change from a CG to a GC closing base pair is also minimal from a sequence perspective; only the location of the C and the G is changing in the unfolded strand. Given that polyethylene glycols have not revealed a sequence preference for their interactions and that the sequence change is small, it suggests that the difference in PEG-based destabilization of the model hairpin loops is not due to sequence differences or interactions in the unfolded state alone.

Previous experiments on GAAA tetraloops have shown an electrostatic contribution for the preference of a CG closing base pair, and the results suggest that potential clashes between the loop and closing base pair are enhanced with a GC closing base pair (Blose et al., 2009). If PEG 200 were exerting its influence based on preferential solvation of the unfolded state alone or via dielectric effects, there should be no change or an enhancement of the preference for the CG closing base pair as the lower dielectric should further stabilize the favorable interactions and destabilize the clashes. This is counter to our observation that 1 cg and 2 cg are destabilized relative to 1 gc and 2 gc. One potential way to explain this observation, however, is to consider the accessible surface area of the loops. For GAAA the loop with the GC closing basepair is less compact than the loop with a CG closing basepair (Blose et al., 2009), and this additional accessible surface area in the presence of a GC closing pair may allow some nucleobase-cosolute interactions to be maintained in the folded state as compared to a loop with a CG closing base pair.

## Conclusion

In this study, we examined the thermodynamics of GAAA tetraloops as a function of the stem sequence, the choice of closing base pair, and the presence of osmolytes. We found that non-nearest neighbor effects do not influence the relative stability of the GAAA loop or the preference for a CG closing base pair. Both PEG 200 and PEG 8000 destabilized the RNA hairpins, but the destabilization was greater than observed for a similar hairpin system with an unstructured tetraloop (Lambert & Draper, 2007). For both PEG 200 and PEG 8000, the extent of destabilization was dependent on the loop closing base pair as GAAA loops with the thermodynamically preferred CG closing base pair were destabilized to a greater extent. Moreover, the free energy gap in loop stability due to the presence of the CG closing base pair is decreased over 40% in 40% (w/v) cosolute. The decreases in thermodynamic preference for the CG closing base pair did not depend on the cosolute size or the nucleic acid polymer as we observed similar results for a DNA triloop. Further experiments revealed that the destabilization of the hairpins as well the decreasing energetic advantage of the CG closing base pair have a linear relationship with PEG 200 concentration. These results taken along with considerations of dielectric effects and similarities of the single-stranded sequences suggest that it is likely that the difference in observed stabilities may be due to PEG 200 interactions with the folded state. Literature observations of increased accessible surface area in the presence of a GC closing base suggest that they may be able to retain interactions with specific cosolutes in the folded structure (Blose et al., 2009). It has been suggested that RNA structures may have evolved to respond to the presence of specific osmolytes (Lambert & Draper, 2007), and it has been shown that cosolutes can promote the cooperative folding of secondary and tertiary structure in functional RNA to enhance stability *in vivo* (Strulson et al., 2014). Our results provide evidence that minimal to non-perturbing sequence changes of even one base pair can significantly influence how cosolutes can influence the stability and folding of RNA structures in *vivo*.

## Additional Information and Declarations

### Data Availability

The following information was supplied regarding data availability: Figshare: https://figshare.com/s/d8422685fe042e323eb2.
